# Allergic Diseases in the Elderly

**DOI:** 10.37825/2239-9747.1046

**Published:** 2023-12-29

**Authors:** Gabriele Di Lorenzo, Marcello Melluso, Alessandro Rodolico, Aurelio Seidita

**Affiliations:** a Department of Health Promotion Sciences, Maternal and Infant Care, Internal Medicine and Medical Specialties (PROMISE), University of Palermo, Palermo, Italy; b General Practitioner, Provincial Health Authority (ASP) 206 of Palermo, Palermo, Italy; c Psychiatry Unit, Department of Clinical and Experimental Medicine, University of Catania, 95123 Catania, Italy; d Institute for Biomedical Research and Innovation (IRIB), National Research Council (CNR), Palermo, Italy

**Keywords:** Elderly, Immunosenescence, Allergic conjunctivitis, Allergic rhinitis, Asthma, Skin diseases

## Abstract

Recent increases in allergic diseases are thought to be caused by better hygiene, Westernized diets, air pollution, climate change, and other factors that influence host microbiota, a key player in the induction and maintenance of immunoregulatory circuits and tolerance. The increase of allergic diseases in the elderly is also related to additional factors, such as various comorbidities that may interfere with the development and the type of allergic reactions. Immunosenescence plays a central role in these reactions, altering microbiota responses and triggering inflammageing. In addition, in the elderly, there is a shift from Th1 to Th2 immunity, thus favoring allergic responses. A better understanding of the mechanisms responsible for immunosenescence and its effects on allergic inflammation will most certainly lead to improved therapies.

## 1. Introduction

### 1.1. Immunosenescence

People aged 65 or older are the fastest growing segment of the population in developed countries, and it is estimated that this group will comprise approximately 20 % of the total population by 2030. The prevalence of allergic diseases in the elderly is estimated to be around 5–10 % [1,2]. Although these diseases are usually known as disorders of childhood and adolescence, allergic diseases often persist into old age and may occasionally make their first appearance in the elderly.

Over the last few decades, environmental and lifestyle changes, including climate change, increased pollution, and modern diets, have affected the saprophytic microbial flora [3]. Variations in the microbiota could be the cause of the establishment of a chronic inflammatory state which triggers and maintains inflammatory as well as allergic diseases [4]. The latter, in the elderly, can be attributed to immunosenescence and to the copresence of other chronic organic diseases. These two overlapping situations can influence the maintenance or onset of allergic reactions [5,6], given that aging is characterized by changes in immunological and nonimmunological mechanisms [7]. In Table 1, we report functional changes in innate and adaptive immunity cells associated with aging.

Thus, there are functional modifications of immune cells and anatomo-functional changes of all organs and apparatuses. These changes ultimately correspond to a decrease in functional reserve.

In this context, certain trace elements, including zinc, iron, and vitamins such as vitamin D, appear to play an important role in the homeostasis of immune responses [8]. However, a relationship between hypovitaminosis D and total IgE has never been demonstrated in the elderly; in fact, IgE levels in the elderly do not differ from those in younger people, whereas they appear to be lower in women who generally have a lower prevalence of allergic diseases [9–11].

Aging mainly reflects the consequence of unrepaired damage over the life course and is characterized by a complex phenotype associated with progressive changes in many organs and systems [12]. In particular, the immune system is profoundly remodeled during aging. Several mechanisms of innate and adaptive immunity undergo age-related changes, resulting in what is known as immunosenescence. This phenomenon is characterized by a progressive decline of some features of immune functioning, while others become more active. The main hallmarks of immunosenescence are imbalances in the lymphocyte subpopulation (a decrease in naive lymphocytes and an increase in memory lymphocytes, with the accumulation of dysfunctional senescent cells with shortened telomeres), thymus involution with a decrease in the generation of new T cells, dysfunction of hematopoietic stem cells [13], defects in apoptotic cell death, mitochondrial function, and stress responses, and the malfunction of immunoregulatory cells. Consequently, a senescent immune system is characterized by impaired interactions between innate and adaptive immune responses, continuous remodeling and narrowing of the immune repertoire due to persistent antigenic challenges, and chronic low-grade inflammation [14,15]. These changes lead to increased susceptibility to new-onset infections and a shift of the immune system towards an inflammatory, autoimmune, and Th2 profile. This immune dysregulation forms the background for the development of increased susceptibility to infections and autoimmune diseases, neoplasms, metabolic and bone diseases, neurological disorders, and allergic inflammation [16].

In 2017, De Martinis elegantly and speculatively described how age-related changes may underlie allergies in the elderly, highlighting how age-related functional and structural changes in the upper and lower airways, skin, and gut contribute to the maintenance and onset of allergic reactions [17].

Despite increasing reports of a rising prevalence of allergic diseases in the elderly, there is a lack of guidelines for the management of allergic diseases in this age group. Demographic changes, with increasing life expectancy, require diagnostic and therapeutic programs specifically dedicated to elderly patients. The progressive aging of the population makes it plausible to predict that allergic diseases in the elderly will have an increasing impact on public health and public healthcare systems in the coming years. Therefore, efforts must be made to correctly diagnose allergic conditions in the elderly and treat them properly.

## 2. Allergic diseases in the elderly

Symptoms of allergic diseases can be limited to one organ or apparatus or be systemic. In this paper, we review several allergic diseases that require allergy counseling and that we have encountered in our clinical practice and which were the subject of previous publications [18–22].

**Fig. 1:**
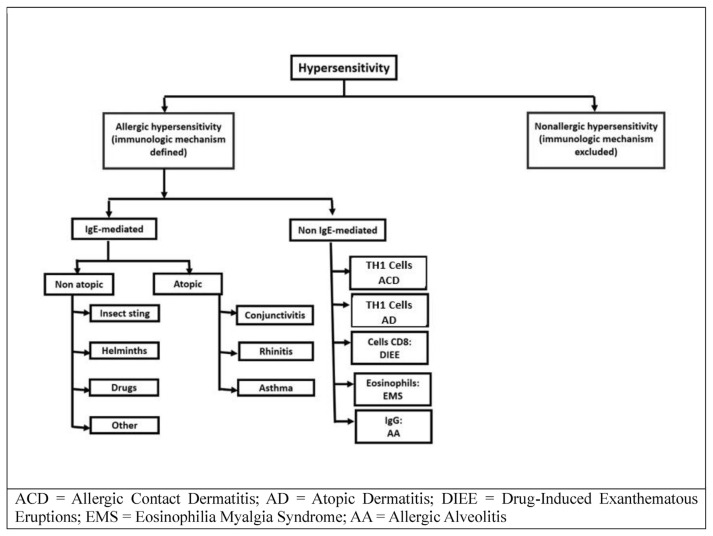
Allergic and non-allergic hypersensitivity diseases.

While we refer to the classic Coombs and Gell classification, we focus only on diseases recognizing type I and type IV immunoreactions, which are the most frequent in clinical allergology practice and which must be differentiated from non-allergic diseases, as reported in the 2002 EAACI document [23]. For this purpose, we have used the scheme reported in Fig. 1.

In Table 2, we report diseases that may recognize an immune reaction and which frequently require allergy counseling [24].

## 3. Clinical features of allergic diseases in the elderly

### 3.1. Airway diseases

#### 3.1.1. Rhinitis

**Fig. 2:**
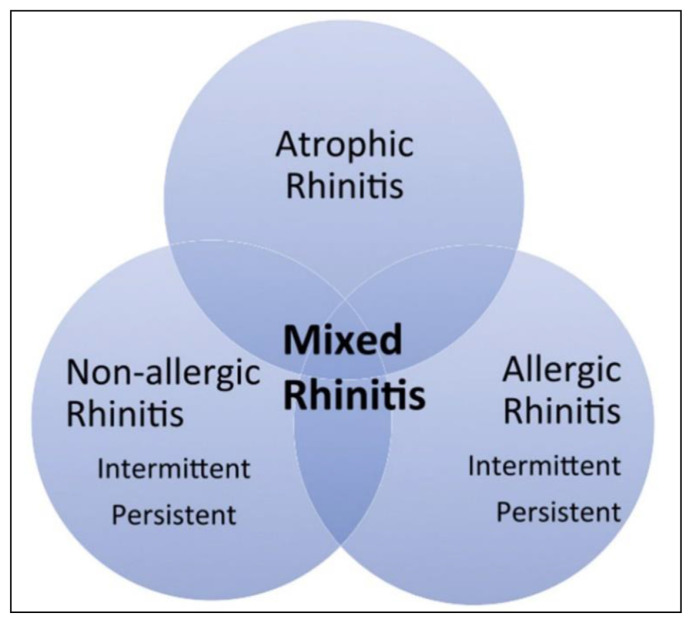
Types of rhinitis observed in the elderly.

Among the diseases that can recognize a type I immune reaction, the most epidemiologically important is rhinitis. Allergic rhinitis (AR) has a prevalence in Europe ranging from 16.9 % in Italy to 28.5 % in Belgium. The prevalence of AR in patients over 65 years of age is estimated to be between 5.4 and 10.7 % [25]. Elderly patients with rhinitis present with the typical symptoms of the disease: sneezing, rhinorrhea, nasal itching, and nasal congestion. Fig. 2 shows the types of rhinitis observed in the elderly as correct diagnosis is important to start appropriate treatment.

In our study, the analysis of data from patients seen in the allergology outpatient clinic of the Department of Health Promotion Sciences, Maternal and Infant Care, Internal Medicine and Medical Specialties (PROMISE) at the University of Palermo (Italy), confirms that allergic rhinitis is less frequent in elderly patients compared to younger ones, but without gender differences. In the elderly, non-allergic rhinitis seems to be more important. Therefore, the diagnostic procedure for rhinitis in elderly patients does not differ from that of younger ones, and includes taking medical history, performing a physical examination, a pneumoallergen-specific IgE assay, and a cytological examination of nasal secretions [18].

#### 3.1.2. Asthma

The Global Initiative for Asthma (GINA) document states that asthma affects elderly patients as much as any other age group, and the prevalence of asthma in the elderly in industrialized societies is estimated to be around 6–10 %. However, mortality is higher in elderly asthmatics than in other age groups, where mortality has been steadily decreasing [26].

**Fig. 3:**
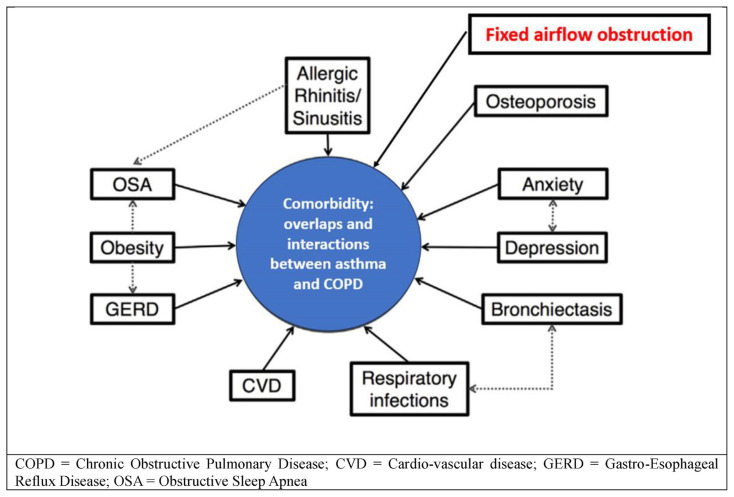
Comorbidity: overlaps and interactions between asthma and COPD.

The diagnosis of asthma is made difficult by some of the problems specific to elderly asthmatics, so much so that it has been estimated that only half of elderly asthma patients receive a correct diagnosis. The comorbidities, overlaps and interactions between asthma and chronic obstructive pulmonary disease (COPD) are shown in Fig. 3, but bronchial obstruction is the real problem [19].

Bronchial obstruction is irreversible in COPD, whereas it is reversible in asthma. In elderly patients with bronchial obstruction, as we have pointed out in a previous paper, correct diagnosis is essential for proper inhalation therapy, with the assessment of the reversibility of bronchial obstruction after a short period of oral steroid therapy. Indeed, bronchial obstructions that seem irreversible are not always so, thus explaining why many elderly asthmatic patients are diagnosed with chronic obstructive bronchitis. A misdiagnosis can cause serious iatrogenic complications as chronic obstructive bronchitis therapy is based on bronchodilators, whereas asthma therapy is based on topical steroids and bronchodilators.

Therefore, the asthma diagnostic procedure includes medical history and a physical examination, along with spirometry at baseline and after using a bronchodilator; if bronchial obstruction persists, new spirometry after a 14-day course of prednisone 50mg/day should be performed. In addition, a blood eosinophil count and cytological examination of induced sputum can assist in diagnosis. A pneumo-allergen-specific IgE assay is almost useless in this context [20].

### 3.2. Skin diseases

The first reason an elderly person consults an allergist is not for an allergic disease itself but rather because of itching: a symptom which, when presenting without skin lesions, is almost never allergic in nature. In fact, pruritus and allergic skin diseases are concomitant to important skin changes in the elderly. There are no significant gender differences in the elderly with itching and allergic skin diseases. Itching, without high skin manifestation, is very frequent in the elderly. However, when assessing an elderly patient with pruritus or chronic urticaria, some systemic diseases that may cause chronic pruritus and/or urticaria must be considered (Table 3).

#### 3.2.1. Allergic contact dermatitis

Allergic contact dermatitis (ACD) is not uncommon in the elderly [27]. The most common contact allergens are nickel sulfate (11–12 %) and balsam of Peru (7–9%). However, topical drugs, such as those used for leg varices, are also a common cause of ACD. Neomycin and corticosteroids can often give late positive reactions [28].

Patch testing is essential for the differential diagnosis of ACD and irritative dermatitis [29].

#### 3.2.2. Atopic dermatitis

Atopic dermatitis (AD), formerly referred to as endogenous eczema, is very rare in the elderly compared to children and young adults. Late-onset AD, without the usual history of atopy, may explain eczema of unknown origin and negative patch tests in the elderly [30].

**Fig. 4:**
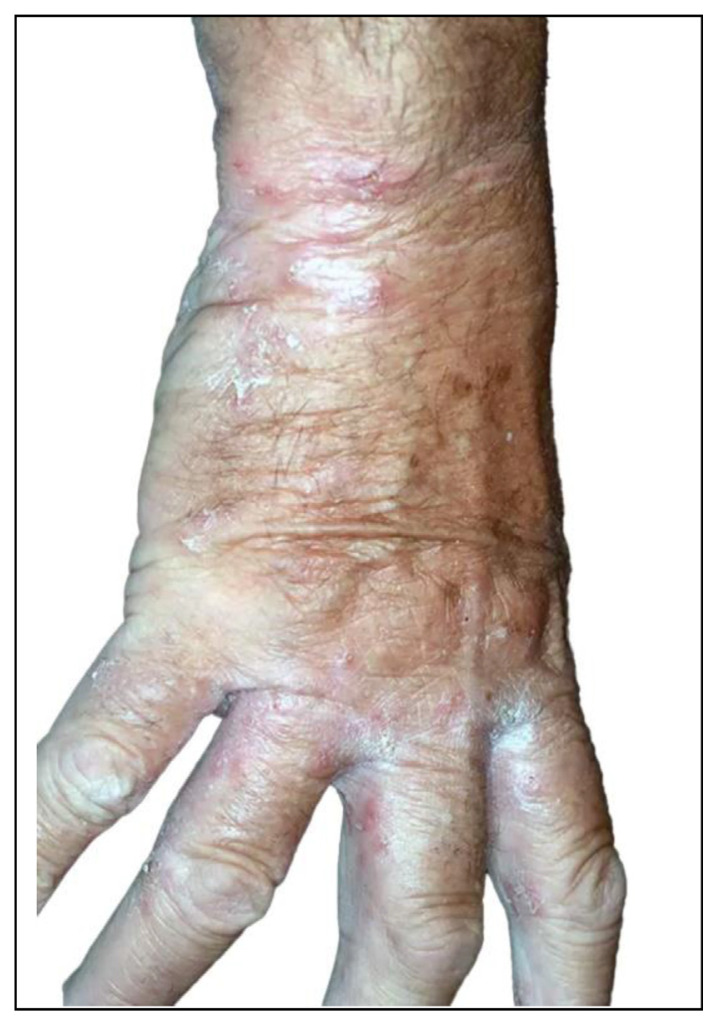
Scabies in elderly subjects with a previous diagnosis of contact eczema.

Scabies must be considered in the differential diagnosis of generalized dermatitis because institutional acquisition of this infestation is common in the elderly [31] (see Fig. 4).

#### 3.2.3. Urticaria angioedema

Urticaria is a common disorder characterized by pruritic weals and/or angioedema. It affects 20–25 % of the population at some point in their lifetime [32]. Chronic urticaria (CU) is defined as the occurrence of weals, angioedema, or both on a daily or almost daily basis for ≥6 weeks. Unlike physical and other inducible urticarias, in chronic spontaneous urticaria (CSU), the appearance of clinical manifestations is spontaneous and not evoked by physical and/or environmental stimuli [33].

In Italy, patients with urticaria are frequently encountered in allergology practice [34].

**Fig. 5:**
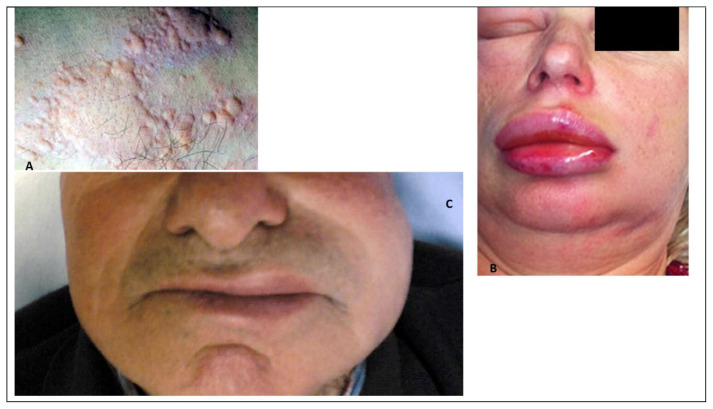
A–C. A: urticaria; B: urticaria and angioedema caused by non-steroidal anti-inflammatory drugs (NSAIDs); C: angioedema caused by angiotensin-converting enzyme inhibitors (ACE-i).

Fig. 5 A–C shows urticaria (see Fig. 5A), urticaria and angioedema caused by non-steroidal anti-inflammatory drugs (NSAIDs) (see Fig. 5B) and angioedema caused by angiotensin-converting enzyme inhibitors (ACE-i) (see Fig. 5C).

CSU, or idiopathic urticaria, is quite common in the elderly, rarely recognizes an IgE-mediated mechanism, and its most pronounced symptom is itching. To make a diagnosis of CSU, the systemic diseases listed in Table 3 must be excluded.

Hereditary angioedema from hereditary C1 inhibitor deficiency (HAE-C1-INH) is exceptional in the elderly, whereas acquired C1-inhibitor deficiency angioedema (AAE-C1-INH) is more common. This is characterized by a massive activation of the classical complement pathway and accelerated C1–INH catabolism, usually due to neoplasms of the lymphatic tissue or autoimmune diseases [35–37].

The prevalence of angioedema from ACE-i is rather high, ranging from 0.1 % to 2.2 %, and should be suspected in all patients with adverse events receiving ACE-i.

Normal levels of complement factors help to strengthen clinical suspicion and exclude the possibility of C1–INH-deficient angioedema.

## 4. Food allergies

**Fig. 6:**
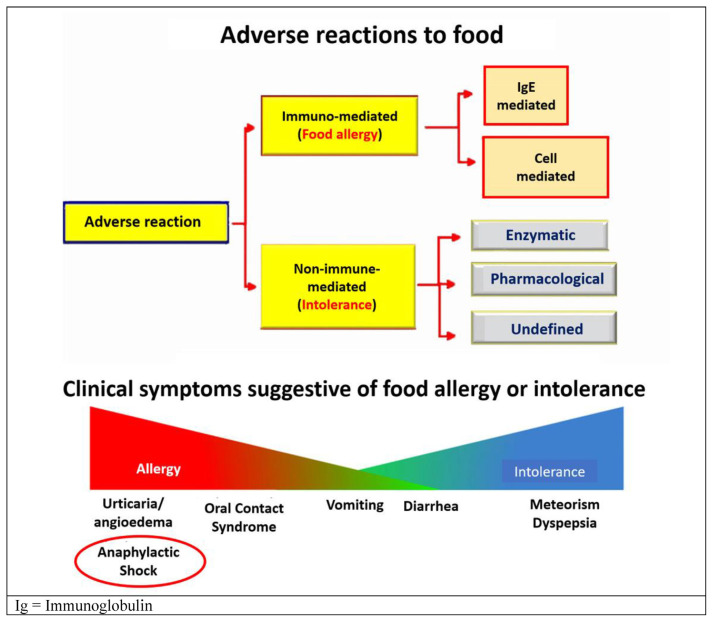
Classification of adverse reaction to foods and clinical symptoms suggestive of food allergy or intolerance.

The issue of food allergies is controversial due to the objective difficulties of diagnosis. The symptoms attributable to a food allergy and/or intolerance are quite varied and include urticaria/angioedema, oral contact syndrome, vomiting, diarrhea, meteorism, and dyspepsia. The most dramatic clinical picture attributed to food allergies is anaphylactic shock which, however, is rarely observed in elderly patients visited in Emergency Rooms (see Fig. 6) [38].

The diagnostic procedures for food allergy diagnosis include: medical history, objective examination, specific IgE assay if one exists for the food allergen indicated by the patient, molecular diagnostic assessment to evaluate the risk of systemic reactions, an elimination diet, and subsequent blind challenge with the food [39].

## 5. Drug allergies

**Fig. 7:**
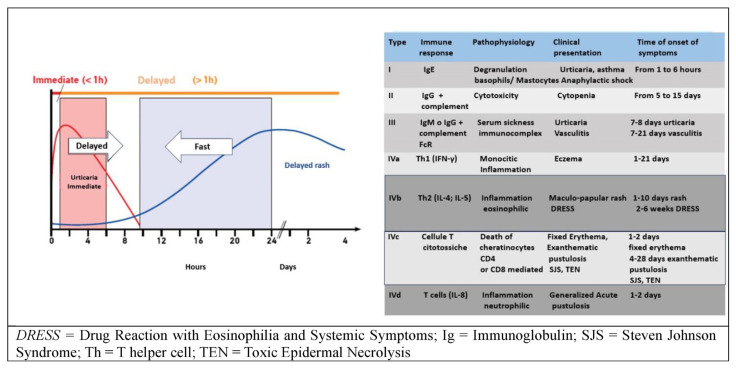
Time relevance for the evaluation of different clinical manifestations.

Allergy to drugs is probably one of the most complex fields of allergology, especially in elderly patients due to the frequency of polytherapy. It is preferable to refer to a drug reaction as ‘hypersensitivity ‘ or ‘adverse reaction’ rather than allergy because the latter term should only be used in cases where an immunological mechanism has been demonstrated. The time criterion, i.e., the time between taking the drug and the onset of the clinical picture, is very important. Allergic hypersensitivity reactions can recognize all immunoreactions of the Coombs and Gel classification, though they have different presentations, pathogenetic mechanisms, and prognoses (Fig. 7) [40].

**Fig. 8:**
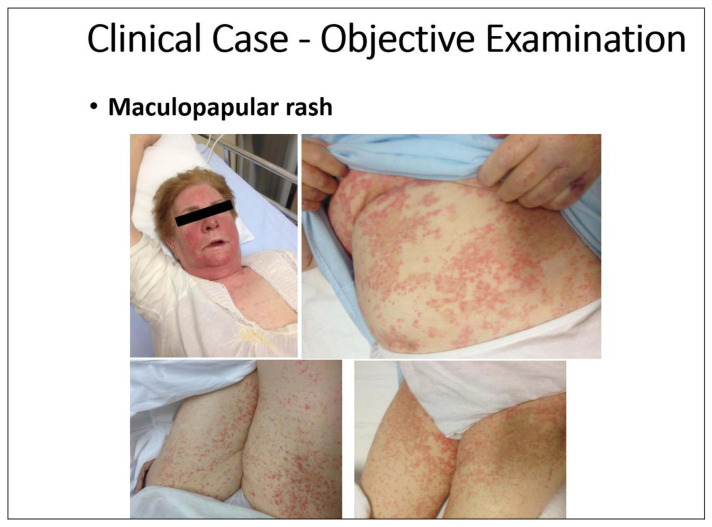
A Case of Fatal Drug Rash Eosinophilia and Systemic Symptoms from Allopurinol [22].

Fig. 8 shows an acute urticaria resulting from non-steroidal anti-inflammatory drugs (NSAID) NSAID use, which is never caused by an immune reaction, and a drug reaction with eosinophilia and systemic symptoms (DRESS) from allopurinol, which instead recognizes a non-IgE-mediated immune mechanism. While the prognosis is good for NSAID urticaria [21], for DRESS it is inauspicious [22].

Therefore, diagnostic procedures must be adapted to the clinical manifestations and the drug suspected to be responsible for the hypersensitivity reaction.

## 6. Conclusions

The only real reason to establish a specific diagnosis for an allergic disease is to prescribe a specific treatment. To date, allergen avoidance and immunotherapy are the only specific treatments we can offer to allergic patients. Pharmacological treatment is only used for symptom control, and no studies have been able to show that the pharmacological treatment of allergic diseases, not even with corticosteroids, is able to change the spontaneous and long-term course of the disease. Consequently, the pharmacological treatment of allergic diseases in the elderly increases the number of drugs to be taken.

Tomorrow’s physicians will have to be prepared to meet the changing health needs of the elderly, considering their peculiar aging-related conditions. Allergic diseases represent an old as well as a new emerging health problem that will have both direct and indirect impacts on patients and on the health care sector. Since many diseases present in ways that are difficult to distinguish from allergic ones, the differential diagnosis of these conditions is crucial in the elderly population.

## Figures and Tables

**Table 1:** Functional changes in innate and adaptive immunity cells associated with aging

Cell type	Changes with aging
Neutrophils	Reduced phagocytosis
	Reduced reactive oxygen species production
	Defect in apoptotic cell death
Eosinophils	Reduced degranulation
	Reduced superoxide production
Mast cells	Reduced degranulation
	Dysregulations in function
Monocytes/macrophages	Reduced phagocytosis
	Reduced cytokine and chemokine secretion
	Reduced generation of nitric oxide and superoxide
Dendritic cells	Reduced phagocytosis and pinocytosis
	Increased IL-6 and TNF-alfa production
	Diminished TLR expression and function
	Dysregulations in function
T cells	Reduced response and proliferation
	Reduced CD28 expression
	Reduced TCR diversity
	Reduced signal transduction
	Dysregulations in function
B cells	Production of low-affinity antibodies
	Increased oligoclonal expansion
	Decline in serum total IgE values
	Reduced surface MHC class II molecule expression
	Dysregulations in function
Epithelial cells	Impaired production of cytokines
	Decreased clearance of particles
NK cells and NKT cells	Reduced numbers or increased in several tissues
	Reduced cytotoxicity and proliferation

**Table 2:** Allergic diseases which frequently require allergy counseling

Rhinitis
Asthma
Contact eczema
Urticaria
Reactions to food
Reactions to drug
Anaphylaxis

**Table 3:** Diseases to consider in elderly patients with generalized pruritus and/or urticaria

Liver diseases	Primary biliary cirrhosis
	Primary sclerosing cholangitis
	Extrahepatic cholestasis
	Hepatitis B and C
Kidney diseases	Chronic kidney insufficiency
Hematologic diseases	Polycythemia vera
	Hodgkin disease
	Non-Hodgkin lymphomas
	Leukemias
	Myeloma
	Multiplex Iron deficiency
	Systemic mastocytosis
	Hypereosinophilic syndrome
	Myelodysplastic syndromes
Endocrine disorders	Diabetes
	Hyperthyroidism
	Hypothyroidism
	Hyperparathyroidism
Neurologic diseases (neuropathic pruritus)	Brain injury/tumor (frequently unilateral pruritus)
	Sclerosis multiplex
	Small fiber neuropathy
Solid tumors (paraneoplasic pruritus)	
Carcinoid syndrome	
Infectious diseases	HIV infection/AIDS
	Infestations

## Abbreviations

ACD: allergic contact dermatitis
ACE-i: angiotensin-converting enzyme inhibitors
AAE-C1-INH: acquired C1-inhibitor deficiency angioedema
AR: allergic rhinitis
AD: atopic dermatitis
COPD: chronic Obstructive Pulmonary Disease
CU: chronic urticaria
DRESS: drug reaction with eosinophilia and systemic symptoms
HAE-C1-INH: hereditary angioedema from hereditary C1 inhibitor deficiency
NSAID: non-steroidal anti-inflammatory drugs
